# A qualitative study to explore experiences of anti-racism teaching in medical residency programs across the United States and subsequent creation of the SPOC (Support - Pipeline - Outcomes - Community) Model to guide future curricula design

**DOI:** 10.1186/s12909-024-05305-5

**Published:** 2024-04-08

**Authors:** Alida M. Gertz, Michele Smith, Davon Thomas, Angeline Ti, Cheryl Vamos, Joe Bohn

**Affiliations:** 1 Wellstar Douglas Family Medicine Residency Program, Douglasville, GA USA; 2 College of Public Health, University of South Florida, Tampa, USA

**Keywords:** Anti-racism, Medical education, SPOC model, Community, Systems thinking, Residency, Curriculum design

## Abstract

**Background:**

Racism contributes to health disparities and is a serious threat to public health. Teaching physicians about racism, how to address it in medical practice, and developing high quality and sustainable curricula are essential to combating racism.

**Objective:**

This study aimed to (1) describe the experience of racism and anti-racism teaching in residency programs, and elicit recommendations from key informants, and (2) use these data and formative research to develop recommendations for other residencies creating, implementing, and evaluating anti-racism curricula in their own programs.

**Methods:**

From May to July 2023, 20 faculty and residents were recruited via convenience sampling for key informant interviews conducted via Microsoft Teams. Interviews were audio recorded, transcribed, and coded. An initial list of themes was developed using theoretical frameworks, and then refined using a grounded-theory approach. A brief online optional anonymous demographic survey was sent to participants in August of 2023.

**Results:**

Eighty percent (20/25) of participants approached were interviewed. Seventy-five percent (15/20) answered a brief optional demographic survey. Seven themes emerged: (1) Racism in medicine is ubiquitous; (2) Anti-racism teaching in medicine varies widely; (3) Sustainability strategies should be multifaceted and include recruitment, resource allocation, and outcome measures; (4) Resources are widely available and accessible if one knows where to look; (5) Outcomes and metrics of success should include resident- faculty-, patient- community-, and system-focused outcomes; (6) Curricular strategies should be multilayered, longitudinal, and woven into the curriculum; and (7) Self-reflection and discomfort are necessary parts of the process.

**Conclusions:**

This study is one of the first to qualitatively examine perspectives of key stakeholders invested in anti-racism teaching for residents. The Support - Pipeline - Outcomes - Community (SPOC) Model, that was developed using information collected during this study, can be used in the future as a guide for others working to design and implement sustainable and high quality anti-racism curricula for residents.

## Introduction

There is no question that racism contributes to differences in health outcomes between different populations and is, therefore, a serious threat to public health [1]. Inequities among different racial groups in the United States are well documented [1–3]. Teaching physicians about racism, learning how to address it within the practice of medicine, and developing sustainable and high quality curricula to do so are key.

Published literature describing anti-racism curricula in residency programs and particularly in primary care programs is growing but still lacking in many respects including data on how to ensure the sustainability and quality of such programs [4, 5]. Curricula that have been described in the peer-reviewed literature include those describing curricular strategies such as lecture series, longitudinal experiences, increasing resident/student knowledge, improving attitudes, decreasing implicit bias, and increasing diversity of residents and staff [6–11].

Although health disparities teaching is now required in family medicine residency programs, it is unclear if and how anti-racism teaching, specifically, is taking place and whether it is effective [12]. Of the small number of social determinants of health post-graduate medical education curricula that exist and are described in the literature, the vast majority do not focus on sustainability or continuous quality improvement efforts [13, 14]. Most reports focus on descriptions of the curricula, evaluation of the curricula and/or residents, and faculty perceptions of and experiences with the curriculum [12]. Similarly, there is a dearth of qualitative research exploring program directors’ and residents’ perspectives of racism and anti-racism teaching in various programs. Finally, few studies have included descriptions of patient-oriented outcomes, which is understandable given the difficulty in demonstrating causation. For example, it is difficult (but not impossible [15]) to design a study that provides evidence that medical students or residents who were included in a curriculum designed to improve empathy, actually went on to care for patients who lived longer due to their chronic diseases being better controlled because of improvements in their providers’ empathy skills [16, 17].

This study aimed to use qualitative data gathered via key informant interviews and formative research to explore approaches to developing a sustainable, and high quality anti-racism curriculum for residency programs in the U.S. Findings from the interviews were used to inform development of a model that programs can utilize as a roadmap or guide when creating, implementing, evaluating, and sustaining and high quality anti-racism curricula in their own programs.

## Methods

### Conceptual framework and model development

The Consolidated Framework for Sustainability Model [4] and Deming’s Theory of Quality Improvement [18] were used to guide the creation of the interview questions and probes*.* A modified grounded theory approach was used to create an initial list of themes, which was then built upon as the transcripts were coded. Using the Consolidated Framework for Implementation Research (CFIR) [19, 20], the themes and sub-themes were then organized to develop a model for development of anti-racism curricula for physicians in training.

### Instrument development

A semi-structured interview guide was developed based on the research aims and the theoretical frameworks described above. Questions were designed to elicit participants' perceptions and experiences with racism in medicine, anti-racism teaching in medicine, and curricular development for residency programs. A brief optional, anonymous demographics survey was emailed to participants to complete after the interviews. The in-depth interview guide is shown in Table 1.

**Table 1:** Questions from in-depth key informant interviews designed to capture experiences of racism in medicine, anti-racism teaching in medicine, and elicit recommendations for strategies to improve and sustain anti-racism curricula

**Question 1:** Can you tell me about any experiences you’ve had with racism in your program? *Probe:* How have these affected residents’ learning? *Probe:* How have these affected patient care? *Probe:* Has your program done anything to address the challenges noted? **Question 2: **Can you describe the extent to which your program discussed or implemented an anti-racism program? If discussed: *Probe:* Can you tell me a bit about how it works? *Probe:* What contributed to the success of setting up and running the program? *Probe:* Can you tell me a bit about what challenges your program faced when designing, implementing, and continuing this curriculum? *Probe:* If challenges, can you tell me a bit about how you overcame, or plan to overcome these challenges? **Question 3:** What other activities has your program implemented to address the problem of racism in medicine? *Probe:* Have these efforts been successful? *Probe:* Why or why not? **Question 4:** How has your organization measured or evaluated the success or impact of the program? *Probe:* Are there any quantitative measures? *Probe: *Are there any qualitative measures? *Probe: *What practical, long-term outcomes have you decided to measure or considered measuring, if any? (For example, financial, patient-centered, resident behaviors, etc.) *Probe:* What sorts of measures, if any, have you developed or considered using to measure whether the curriculum is having the desired downstream effects on things like resident behaviors in practice, and changes in the health of the patients and/or communities your residents serve? **Question 5: **In what ways have you incorporated quality improvement efforts into your curriculum development and/or ongoing activities (if any)? **Question 6:** In what ways (if any) have you tried to make the curriculum sustainable? *Probe:* Have you considered actions at the program level? *Probe:* Have you considered actions at the health system level? *Probe:* Have you considered actions at the community level? *Probe:* Have you considered activism at the state/federal policy level? *Probe:* Any staffing or recruitment considerations? *Probe: *What sorts of resources are needed to create a sustainable program? **Question 7: **What advice do you have for other programs thinking about designing and implementing an anti-racism curriculum for residents? Or what would you imagine would be included in an ideal anti-racism curriculum? *Probe:* What would this sort of curriculum look like? *Probe:* What resources would you include? *Probe:* What outcome measures would you look at? How would you measure success? *Probe:* How would you make it sustainable? *Probe:* How would you implement quality improvement efforts?	

### Sampling and recruitment

A convenience sampling technique was used for recruitment. Participants included faculty and prior residents from across the U.S. from multiple specialties, who had either thought about or been involved in designing, implementing, and evaluating anti-racism curricula in their own institutions. We included not only faculty but also residents and former residents who had been the recipients of such efforts or lack therof. Subjects were recruited via emails, texts, or in person conversations. Participants were chosen based on prior knowledge or pre-existing interest, experience, and/or expertise in the area of study, and the lead author’s or colleagues recommendations. Participants were invited to interview until theoretical redundancy was reached. Inclusion criteria were (1) adults age 18 years or older who (2) worked at a residency program, were current residents, or had previously been a resident in a residency program in the U.S. No incentives were provided. Participation was voluntary. Verbal informed consent was obtained from participants prior to involvement in the study. A concerted effort was made to ensure that at a minimum half of all participants were those who identified as under-represented minorities.

### Data collection and analysis

The lead author with expertise in public health, clinical care, and medical education utilized the in-depth interview guide to conduct, record, and transcribe the interviews via Microsoft Teams from May to July 2023. Interviews lasted on average approximately 40 minutes (range 20 minutes to 2.5 hours). Transcriptions were de-identified and saved in Microsoft Word and then collated and coded in Microsoft Excel. A brief online optional anonymous demographic survey was sent in August of 2023 to participants who completed the interviews. Data from the in-depth interviews were analyzed using coding and thematic analysis. AMG developed the initial list of questions and themes; JB, MS, and MV reviewed and provided feedback. AMG then conducted and transcribed the interviews using the updated questions. Prompts were updated to include subthemes that arose during previous interviews. Coding was initially conducted by AMG with review and adjustment by MS and JB. Themes, sub-themes, and representative quotes were initially compiled by AMG with input and adjustment based on feedback from MS, JB, DT, and AT. AMG developed the SPOC model (described below) and refined it based on feedback from all of the other authors. The final list of themes and the model were reviewed, updated, and approved by all authors.

## Results

Of 25 potential participants approached, 20 (80%) completed in-depth interviews and 15 of those 20 (75%) completed an additional optional post interview brief demographic survey between May and August of 2023. Interview participants included faculty, current residents, and program directors. Participants included people who self-identified as persons of color, as well as those who self-identified as being part of other minority communities (e.g., religious minorities and sexual minorities). Geographic location of participants also varied but included participants from the East Coast, South, Mid-west, and Western U.S. Details of the demographics for those participants who opted to answer the brief demographic survey questions are shown below in Table 2.

**Table 2:** Demographics of respondents to in-depth interviews discussing anti-racism curricula for physicians in training, who opted to also complete the brief post-interview, anonymous, online survey

	Number^a^	Percent
Age group (*n* = 15)		
18-35	2	13
36-64	13	87
65+	0	0
Gender (n = 15)		
Male	5	33
Female	10	67
Other	0	0
Practice location? (n = 15)		
West coast of the US	3	20
Northeastern US	6	40
Southern US	4	27
Mid-western US	1	7
Outside the US	1	7
Current trainee? (n = 15)		
Yes	0	0
No	15	100
Currently involved in formal teaching? (n = 15)		
Yes	11	73
No	4	27
Part of an underrepresented group in medicine (e.g., racial, sexual, religious minority group)? (*n* = 14)		
Yes	7	50
No	7	50

^a^Data missing for 5 participants

Seven major themes emerged from the interviews and within each major theme, a number of sub-themes emerged. The major themes that emerged were: (1) Racism in medicine is ubiquitous; (2) Anti-racism teaching in medicine varies widely; (3) Sustainability strategies should be multifaceted and include recruitment, resources allocation, and outcome measures; (4) Resources are widely available and accessible if one knows where to find them; (5) Outcomes and metrics of success should include resident- faculty-, patient-, community-, and system-focused outcomes; (6) Curricular strategies should be multilayered, longitudinal, and woven into the curriculum; and (7) Self-reflection and discomfort are necessary parts of the process. 

### Major theme 1: experiences of racism in medicine are ubiquitous

Subthemes included in the first major theme regarding ubiquitous experiences of racism in medicine highlighted that everyone has either witnessed and/or experienced racism directed at themselves, their colleagues, and/or their patients. Some have experienced extensive racism and had little recourse. One respondent noted, “I can say that at all stages of my training I experienced racism,” while another recalled, “One of the attendings … asked me about my visa status...” (from someone who was born and raised in the US). Another noted, “I frequently got mistaken for [the one other dark-skinned person in the program]” Another subtheme was that outright racism was not uncommon and ranged from use of the “N word,” to assuming a physician was someone on staff with lesser training such as a student, environmental services staff, transport staff, nurse, or tech. One participant recalled, “One of my attendings … would always wear a suit and he was the only black attending that I can recall in the ICU ... A lot of the other attendings would wear scrubs … and when he was asked by one of my co residents... ‘why do you always wear suits, including on weekends?’, he said, ‘So that people take me seriously.’” Another subtheme was that racism exists on a spectrum ranging from micro-aggressions (in written word, spoken word, and actions and gestures) to structural and systemic racism such as policies and social/cultural “norms.” One respondent noted, “I had an attending who would joke about me being from Africa and [ask] if I swing trees or have pet lions.” Another subtheme that emerged was that racism exists in the medical system in multiple forms including but not limited to race-based medicine and calculators to expectations of outcomes based on race to misguided attempts to address racism. Highlighted here was also the point that racist policies instituted by local, state, or federal government (such as restrictions on use of the words equity and diversity) all the way down to racist clinic policies (such as allowing physicians to refuse to see patients who arrive >10 min late) exist and contribute to the problem. To illustrate this, one responded noted, “There are a lot of things [we could do to improve care for our under-represented minority patients, for example], if patients arrive late, you can accommodate [because] you know they obviously have struggles... a lot of the time [they have] transportation issues or [other barriers] we just don’t know [about].” Several people discussed discrimination based on other characteristics (e.g. sexuality, religion, etc.) and the importance of considering intersectionality in such curricula. Tokenism was also mentioned, and it was noted that often people of color are pushed into equity and anti-racism work even when they may prefer to focus on other things. When they are interested in doing this work, they are often poorly compensated. One participant recalled, “I’ve had promotions announced [and then] people comment that it was smart of my boss to [promote me] because [they could now] ‘check the diversity box.’”

### Major theme 2: experience of teaching anti-racism in medicine are variable

Multiple subthemes were elucidated as part of the second major theme exploring experiences of teaching anti-racism in medicine. Multiple participants said they had little to no anti-racism teaching in their own training. Some reported they had a minimal amount, but it was named something else such as “social determinants of health” or “cultural competency.” One participant recalled: “I can remember maybe once or twice where there was some discussion within dermatology specifically about different appearances of rashes on darker skin, and that was the extent of it, though. There was absolutely no discussion of racism specifically or its impacts on health.” Another recalled, “My medical school and residency both had lectures and seminars on cultural competency, [but] I can’t remember anything... like an antiracist curriculum at any point.” One subtheme that emerged here was that the tone for anti-racism teaching is set from the top. System leaders, chairs, faculty/program directors must be an example and set the tone for anti-racism action and teaching. One respondent noted, “I really try to model my own style around those [attendings] that I came to respect very highly … to be able to confront the [racism] issues directly.” Many respondents reported at least attempting to engage in teaching anti-racism in their current practice. Some had very developed curricula; several even reported enacting other anti-racism measures focused on recruitment and retention efforts, and redesigning activities that previously perpetrated implicit bias (such as interview selection rubrics, orientation activities, and even morbidity and mortality (M&M) presentations). Representative quotes describing development of such curricular improvements to address racism included: “The ambulatory attending rounds were not infrequently centered around issues of race and implicit bias.” And “We received a grant ...to create a virtual reality application intended to teach providers about social determinants of health and Health Equity, which is to say also … about racism... I led a team that created [the] exercise ...[that] dropped the user into [an] … under-resourced neighborhood and has them follow a family through six different scenes [describes each scenario]. [Learners are then] assigned to look for help assets and health risks within the setting.” Participants reported in their current programs, having a number of activities aimed at educating residents about equity including didactics, interactive sessions, case-based learning poverty simulations, outside consultants, and support groups for underrepresented minorities (URMs). Finally, a few respondents noted that their programs really emphasized collaboration with the community and having community representation.

### Major theme 3: sustainability considerations for anti-racism curricula should focus on recruitment & retention, resources, and systemic changes

Subthemes noted within the third major theme of sustainability efforts included the importance of integrating anti-racism outcome measures into credentialing, accreditation, and other metrics needed for individual or program success (such USMLE, MCAT, specialty board exam questions, ACGME core competency, and hospital equity scores tied to grant funding). Representative quotes highlighted the importance of having “measurable outcomes in the population that residents treat, [including things like] satisfaction scores.” One respondent said, “Every ACGME accredited program has specific milestones that … are connected to EIDA... I would like to see that happen on a specialty-specific basis. I would like to see that at the ACGME level, it’s happening through the clinical learning environment review. Healthcare disparities are there as part of healthcare quality but don’t really have teeth behind them. So, to make it an ACGME requirement, to really add in that language in the different core competencies, would be something that could be really powerful.” Another commented, “I think there should be anti-racist and implicit bias [criteria included in] how medical students are evaluated. … [and] when you apply to medical school, it should be part of the screening... it ought to be *as* important as all the other things that you screen for.” Another subtheme that emerged in the sustainability category highlighted the importance of ensuring all lecture topics covered during residency include teaching points on equity and anti-racism. One respondent said anti-racism should be “...more integrated in every topic, every talk... if we’re talking about communication, [we mention] cultural humility. If we’re talking about heart failure, [we note] healthcare disparities in this area. So it is really woven into everything that we’re doing instead of set aside as its own topic.” Ensuring dedicated staff, with protected and compensated time, are in charge of leading the effort, while also ensuring buy-in and an ownership feeling by all, was another subtheme articulated. Participants said, “[You] not only need funding, but [also] to have a strong commitment.” And that “having that ownership and pride from the people higher up is paramount.” The significance of finding and ensuring ongoing and permanent resources and support are present and sharing training opportunities across the system was also highlighted. Interviewees noted, “[Programs should capitalize on] any ways in which you can combine forces, because obviously these concepts are transdisciplinary, so there’s no need for each program to recreate the wheel and there’s power in numbers if multiple programs in the same institution can have a shared curriculum.” And “Many of our educational leaders have become trainers as well of those programs. So not only are they receiving the training, but they’re providing the training so that there’s some built-in model where we have local expertise.” Ensuring URM are recruited, supported, and promoted to leadership positions, and providing extra mentoring and support for URM, was another subtheme that emerged. One participant noted, “…it’s not just limited to finding someone who was previously not going to get in or get selected and bringing [that person into your program] … [this can’t undo] the decades or centuries of suppression … it is more likely that people from those groups are going to have struggles and those struggles need to be supported as well. So we had systems for making sure we provide [support].” Another subtheme that emerged was promoting STEM in local communities to encourage kids from URM groups to be interested in medicine. One interviewee commented, “One of the structural things that is currently being done... is to make sure that when we are looking at hiring program directors, assistant program directors, or core faculty, that we are thinking about diversity, and if we don’t have representation from groups historically underrepresented in medicine, then we’re probably not trying hard enough to sponsor and mentor faculty members to aspire to those roles.” The importance of continuous quality improvement efforts was also highlighted. Finally, it was noted that changing the mindset from “We are doing this to help URM,” to “We are doing this to make us all better,” was noted to be very important.

### Major theme 4: tools for curriculum building are abundant if you know where to find them

Subthemes in the fourth major theme focused on different types of resources and strategies available to build curricula and included: books & podcasts and having residents present on a topic they learned about listening or reading, using problem and case-based learning (e.g., Cases that explain topics such as the roots of health care disparities or unfair consequences/discipline for similar actions in white residents vs. URM), using online simulations and other tools, inviting speakers, having pre-recorded lectures, having paid expert consultants who may be URM themselves, inviting speakers from the community or creating a community medicine rotation, shadowing other staff in clinic or hospital, didactics, and having colleagues share their own experiences. One respondent noted, “Having small groups [where] people just like you ... [including] leaders [can openly] discuss … experiences [during] residency, [including those involving] racism, or homophobia, [etc.,] ... getting people’s personal perspective... allow[ing] people to be comfortable, [especially for some white residents/students who] might be sheltered … it might open their eyes [or allow them to realize] colleagues … have experienced all these horrible things .... I think would be really useful because in a sense... talking about personal experiences [will help people understand] … then you won’t have those people who [say] ‘racism doesn’t exist’ or ‘it doesn’t happen anymore.’ When you hear about it firsthand from your colleagues. I think that really makes a huge statement.”

### Major theme 5: outcome measures should be multilayered and include resident, faculty, patient, community, and system metrics

Subthemes in this major theme about outcomes, highlighted the importance of using varied metrics to measure curriculum success. One technique mentioned use of resident pre-post surveys/tests/interviews (to measure knowledge, attitudes, and beliefs). Use of the Kirkpatrick Model for curriculum development, was also mentioned. Respondents also commented on specific strategies used in their own programs: “[To capture] people’s perceptions of the talk itself, whether they intend to change their practice ... surveys are offered right after the talk is given, by email.” Another noted, “Residents take the implicit bias test every so often,” and another noted use of a “test or quiz … to reflect whatever knowledge was gained during that portion of the curriculum,” and yet another commented on the usefulness of “a group discussion .. making sure that they’re applying the correct knowledge and guidelines to patients.” One respondent also suggested, “Assess … residents of color. How comfortable [are] they … with the curriculum and ... how comfortable are they addressing their [program leadership] if there are issues, or if they’re being discriminated against. How confident are they about [being able to successfully] seek help [or] discuss those issues [with program staff, faculty, or leadership]?” Demographics tracking (such as the percent of URM students considered for interviews, the percent matched, the percent that go on to become faculty, and the percent that pursue fellowship) was also emphasized as an important metric. One respondent commented, “The first thing is … keeping track of … how many [URM] residents we had in our program,” and another noted the importance of “whether or not the number of URM residents and faculty had increased over the years to have ... racial concordance to match our patient population,” and yet another suggested tracking “...how many residents of color have we admitted... how many of them have [completed the] program...” One participant said, “...if your intern classes every year have a high representation of black male residents, but those residents are all not completing the program, then that’s obviously a problem.” Another suggested, “Track the number of residents who self-identify as underrepresented in medicine who applied to our programs, who interview at our programs, and who get ranked.” Another subtheme in this area included tracking patient-oriented outcomes such as use of patient surveys, and measuring patient access to care, and patient health outcomes. One participant advised looking at measures such as “[Are] patients not showing up for follow up visits? [Adhering to] medications? [Are] patients actually connected with care? [Do they] trust in the care that they’re receiving or trust the doctors that they have?... If ... not … why?” Another suggested “seeing if your community feels that we are doing everything in our power to make sure that people are treated equally and fairly,” and a third suggested, “[Look at URM patients compared to white patients. Are they] getting their screenings? [Having] improvements in their A1C?” Tracking system resources and funding allocation and hiring equity outcome experts as consultants were other subthemes that emerged.

### Major theme 6: curricular strategies should be multipronged, include integration, and be longitudinal

Subthemes surrounding curricular design strategies highlighted that integration into all other parts of the curriculum should occur, that longitudinal integration was needed, and that it was important to provide evidence and data, when available, to support teaching. It was noted that there is a vast literature on the health effects of racism and it is important for educators to use it to teach residents. Incorporating personal stories of experiences of racism in the medical system faced by patients as well as medical professionals was also suggested. An anti-racism journal club was recommended by several participants. Having a safe space for discussion and also a venue to provide anonymous feedback without fear of retribution was noted as an important curricular strategy. One respondent suggested, “[Have an] anonymous comment box where people can feel free to speak up about topics without having their name associated with [their comments],” while another pointed to the importance of “developing openness in your program that allows for the residents and other team members and patients - the full community at the residency program - to be able to share perspectives.” A broad range of “what not to do” recommendations were made and comments in this subtheme included: “You can’t just hire one tokenized black person with no budget, no protected time, no equitable advancement, or promotion [potential],” and when discussing a part of residency where residents would visit a poor neighborhood adjacent to the hospital one responded noted that they “... don’t know if the word is voyeuristic, but it initially had been very problematic in the sense that it just felt more like [we were] touring this place as opposed to actually learning about the history and integrating ourselves [into the community].” And (referring to the online system-wide required ‘bias’ courses) another noted, “… at least from my experience, most people just check them off or don’t really pay that much attention.” And “…required training course that you do online that you have [to complete to get] your hospital privileges or clinic privileges, like … an EHR training, and I felt that was really useless because most of the time people just very quickly click through them…” Another commented: “We implement systems, and nobody really cares about any form of evaluation..“ Finally, it was emphasized that programs must come up with unbiased ways to deal with “concerns” raised about residents of color to ensure all concerns were dealth with fairly. Finally recognizing that URM residents might not always feel safe speaking out when they have questions, was noted as an important step in creating an effective learning environment.

### Major theme 7: self-reflection, discomfort, engagement are all necessary

The final major theme around introspection and advocacy encompassed a number of subthemes including (1) a need to address and advocate for change on a social level and in society at large, not just in medicine, (2) recognizing that many of the murders that occur are traumatic for URM residents, and (3) addressing potentially unrecognized or uncomfortable issues such as white fragility, and ensuring each person engaging in this work should start by critically examining their own beliefs and biases. Another point that was noted was that strategies that work for programs based in urban academic centers, might not work for more rural programs. Likewise, programs with many IMGs might need to consider a slightly different approach from programs with mostly US medical graduates. One participant (from a program with many IMGs) said, “... I have talked about colonialism and how we also can translate that to how our black population in the US has been treated historically, but not everyone seems to have that same connection ... I have had residents tell me ‘I grew up in [X country], no one has anything. Everyone suffers. Poverty is everywhere’ .... Many of them have told me ‘When I come to the US, I assume this is the best health care system in the world ... so why isn’t this patient taking their meds?’ So much of our curriculum content is geared towards US grads - I find it doesn’t always fit what I need for my [residents].”

A full list of themes, sub-themes, and additional select representative quotes are shown in Table 3 below.

**Table 3:** Themes, sub-themes, and selected quotes from in-depth key informant interviews describing experiences of racism in medicine, anti-racism teaching in medicine, and strategies to improve and sustain anti-racism curricula

Theme	Sub-theme	Selected quote(s)
1) Experiences of racism in medicine are ubiquitous.	A) Everyone has either witnessed and/or experienced racism.	“I have so many examples, we would honestly need to interview for weeks or months on end.”
	B) Outright racism was not uncommon.	“When I was called the 'N word' by a patient, they changed them to another provider, nobody followed up with me.”
	C) Racism exists on a spectrum.	“I’ve seen [hospital] managers treat [staff] differently based on race, like having a different standard for certain … tasks.”
	D) Racism exists in the medical system in multiple forms.	“The structural racism that is inherent in medicine I think is really important to acknowledge as well. Things like different values for GFR for patients with chronic kidney disease [based on race]. That’s an example of structural racism, where the system is … saying that a lower GFR is less serious in a [black] patient than in a white patient.”
	E) Racist policies exist and contribute to the problem.	“We officially are not allowed to say the word ‘diversity’ anywhere in our documents.... Nothing [with] state funding can have the D word in it.”
	F) Important to consider intersectionality.	“[A patient} asked me if I was Jewish and [said] they wouldn’t want a Jewish doctor because of whatever various anti-Semitic ideas they had, and I did have [other] encounters with patients who were either neo-Nazi or [part of] the KKK, but there was no specific support structure around that, and I didn’t report it to anybody, I just carried on with my duties as a resident.”
	G) Tokenism contributes to the problem.	“...the oppressed are usually being put in the position and tokenized to fix the problem on behalf of the oppressing system.”
2) Experiences of teaching anti-racism in medicine are variable.	A) Lack of anti-racism teaching is common.	“There wasn’t any education provided or any training provided in our curriculum that pertain[ed] to racism.”
	B) The tone for anti-racism teaching is set from the top.	One participant noted that when patients made racist comments during rounds, “[as a] more a junior person on the team, you always look to your attending or your senior resident ... to see what their reaction is and often [that] sets the tone for the rest of the encounter.”
	C) Most respondents reported at least some engage in anti-racism efforts in their program.	“There’s been a huge effort both at the residency and faculty level in recruiting trainees and faculty who are historically underrepresented in medicine and supporting [them] once they’re there.”
	D) Participants reported a range of activities across their programs & institutes.	“Our residency lives within a larger Medical Center that has a robust equity inclusion and diversity [program that] puts on events for the entire Medical Center quite regularly.”
	E) Some emphasized community collaboration and representation.	“They oftentimes will have a community or patient representative so that they have a voice [in program planning and design].”
3) Sustainability considerations for anti-racism curricula should focus on recruitment & retention, resources, and systemic changes.	A) Integrate outcome measures into credentialing & accreditation	“There [are] some outcomes ... like testing scores or step scores [where anti-racism knowledge could be tested].”
	B) Ensure all lecture topics covered include a slide on equity.	“...we provide a PowerPoint slide showing an example of a framework that they can use and that has been really nice and ... so even if they’re talking about OB... or GI, then [disparities or racism] components can be included.”
	C) Ensure dedicated staff with protected and compensated time.	“...having a champion, someone in the program who will continue to help lead it and to make sure that it’s integrated year after year into the curriculum.”
	D) Ensure permanent resources and support are present.	“[Hire] someone to specifically look at diversity and inclusion.”
	E) Ensure URM are recruited, supported, and promoted.	“... this training pathway is very complicated and what you have to do, to get into medical school, to get into residency, to be successful, [is difficult]. The training is not easy, and having somebody who can coach you through [all of this] is essential... [providing this] kind of mentorship connections at much earlier stages [is necessary].”
	F) Promote STEM in local communities.	“[Encourage staff to support elementary and high school] students from historically under-represented minorities in STEM [programs].”
	G) Consider additional training tracks for residents and/or faculty.	“[An equity fellowship supporting an annual] cohort of 30 to 40 fellows and residents from all across different departments [who participate longitudinally to learn about equity].”
	H) Change mindset from “We are doing this to help URM,” to “We are doing this to make us all better.”	“We [are] moving away from [saying] ‘let’s bring in trainees [from] under representative minorities for diversity’s sake,' to ‘it makes us a better program, a better healthcare system, because there is just so much added benefit to having our workforce represent the communities that we’re serving and to have diverse viewpoints...’”
	I) Continuous quality improvement efforts are needed.	“[Another important] piece of this of course was the quality improvement piece where we tried to actualize an intervention to try and reduce disparities.”
	J) Create equitable ‘scoring rubrics’ for resident selection.	“When we were looking at our process, we realized that there were a lot of issues … [for example] when we asked our current residents [what sorts of questions they were asked in interviews, we found] a lot of applicants and residents were picked or ranked highly [based on] having more similar hobbies as opposed to clinical skills or life experiences that were important … Now we [use elements of] our program mission and values [in] deciding what to score ... for example [skills related to] clinical medicine, social medicine, and community medicine.”
4) Tools for curriculum building are abundant if you know where to find them.	A) Have residents present on books and podcasts.	“[Have residents review] podcasts, print media, videos, [etc., and then assign them to give] their own presentation [to faculty and co-residents on a topic they choose in anti-racism or disparities].”
	B) Use problem and case-based learning.	“[Have residents watch and evaluate [case scenarios].”
	C) Use online simulations and other tools.	“A poverty simulation ... on[line] that you can walk through, and you you have X amount dollars, and you have to make decisions [about feeding your kids vs taking medications etc].”
	D) Invite and compensate speakers or consultants who are URM.	“There’s a bunch of [URM] people that they own their own [consultant] companies [that can support anti-racism work].”
	E) Invite speakers from the community.	“We .. had.. patients talk to us about their experience with racism in the hospital setting... they [shared] their perspective, what they go through when they walk in the door of the hospital [and it was eye opening].”
	F) Shadow other staff in clinic/hospital.	“Integrate residents into working with our chaplaincy service [and have them work] with our transitional care team to try to understand the barriers that our patients face.”
	G) Have colleagues share their own experiences.	“I really believe in the power of testimony. So listening to either minority patients or minority physicians talking about what their experience has been ... that perspective is really useful.”
5) Outcome measures should be multilayered and include resident/faculty, patient, community, and system metrics.	A) Resident pre-post surveys or tests	“Test or quiz … to reflect whatever knowledge was gained during that portion of the curriculum.”
	B) Demographics tracking (%URM considered for interviews, matched, faculty, fellowship)	“ . . . whether or not the number of URM residents and faculty had increased over the years to have ... racial concordance to match our patient population.”
	C) Patient surveys	“Ask the patients to... to fill out a survey that says, hey, how did you feel about this?... Did you feel like your needs were met? [Then track responses over time]”
	D) Track patient access & health outcomes.	“Are minority patients having shorter visits than white patients?... Getting more diagnosis codes that are associated with things like malingering or drug seeking behavior?... [Look at] data points like the way care is delivered … whether it’s effective care.. [do] they [get] preventive medications? How many calls it takes to reach a physician? … All these sorts of access to care, metrics... You could see how those metrics change over time, particularly with the URMs.”
	E) Track system changes such as resources allocation and fundings.	“You’re not serious if it’s not embedded into the priorities of your strategic plan, if it’s not embedded with a person who’s actually hired, actually empowered by protected time and money, and if there aren’t evaluation systems to [ensure] this is happening... [You’re not serious if you’re] not giving them the same things that we give traditional researchers and predominantly white people in academic spaces.”
	F) Hire equity/disparity outcome metrics consultants.	“There are black ... or people of color who have spent years training and building entire business[es] around antiracism work… budgets must be made to pay those people.. the same way as hospitals ... pay millions of dollars for other things, they need to invest in this.”
6) Curricular strategies should be multipronged, integrated, and longitudinal.	A) Integration into all parts of the program	“Something that starts in orientation and continues longitudinally throughout the program is really helpful.”
	B) Provide evidence and data to support strategies.	“Physicians respond to data and numbers... [so you need to include this in] presentations.”
	C) Incorporate personal stories.	“It’s not real until they actually see a person and they’re like, hey, you know, this is what happened to me.... Someone who already has some social capital so that they felt like they could speak in this space... another physician [to] talk about their experience as a patient or their experience … [of] racism so that they humanize it.”
	D) Create an equity journal club.	“A good ... anti-racist library and journal club, [can be] really effective.”
	E) Include a broad range of topics.	“...other components that factor into healthcare would include housing discrimination, the criminal justice system, and even components of reproductive justice, which has been a very important talk about.... social political climate, and also... what’s happening in their own specific [city/town/state].”
	F) Open safe space and allow for anonymous feedback.	“Develop that kind of space where people could discuss things as they come up is pretty important, and … [utilizing] a trauma informed [approach] where people can contribute from all levels.”
	G) A broad range of “what not to do” recommendations were made.	“...these one-time events [like posting a] mission statement... on a website, and that’s it.”
	H) Come up with unbiased ways to deal with “concerns” raised about residents of color and recognize that URM residents might not always feel “safe” speaking out when they have questions.	“[as a white male] I always feel confident and comfortable enough to say I don’t understand that or I can’t figure it out [but URM trainees might not feel they have the privilege or they will be penalized more harshly or perceived as incompetent].”
	I) Consider creating safe “meeting” spaces where trainees of color can discuss their experiences with one another.	“... a group that was formed for minority students and they got a separate room, separate tutors, a space where they could talk about their experiences”
7) Self-reflection, discomfort, and engagement are all necessary.	A) Advocate for change on a societal level.	“There’s so much work to do and we’re barely scratching the surface with what we are doing..."
	B) Address uncomfortable issues such as white fragility.	“[as a white person sometimes] you have to be uncomfortable, have to get to a place where you feel uncomfortable to grow.”
	C) Each person should critically examine their own biases.	“... you know, I don’t believe I’m a racist and probably most people in medicine don’t, and yet we have these differing outcomes [about] which we have to be honest with ourselves … what can we do to bridge the gap?”
	D) Strategies that work for one type of program might not work others.	“... academic centers are often urban and almost always large well run systems ... when you try to take that same idea and move it to a [smaller more rural system] the concepts are very different, and the people are very different. If you don’t consider [this, then your efforts may] fall short.”

Specific resources such as books, book lists, articles, simulation websites, consultants, podcasts, and other tools were collected during the course of this study, and can be found here: https://www.medicalantiracismcurriculum.com/.

### Model development

**Fig. 1:**
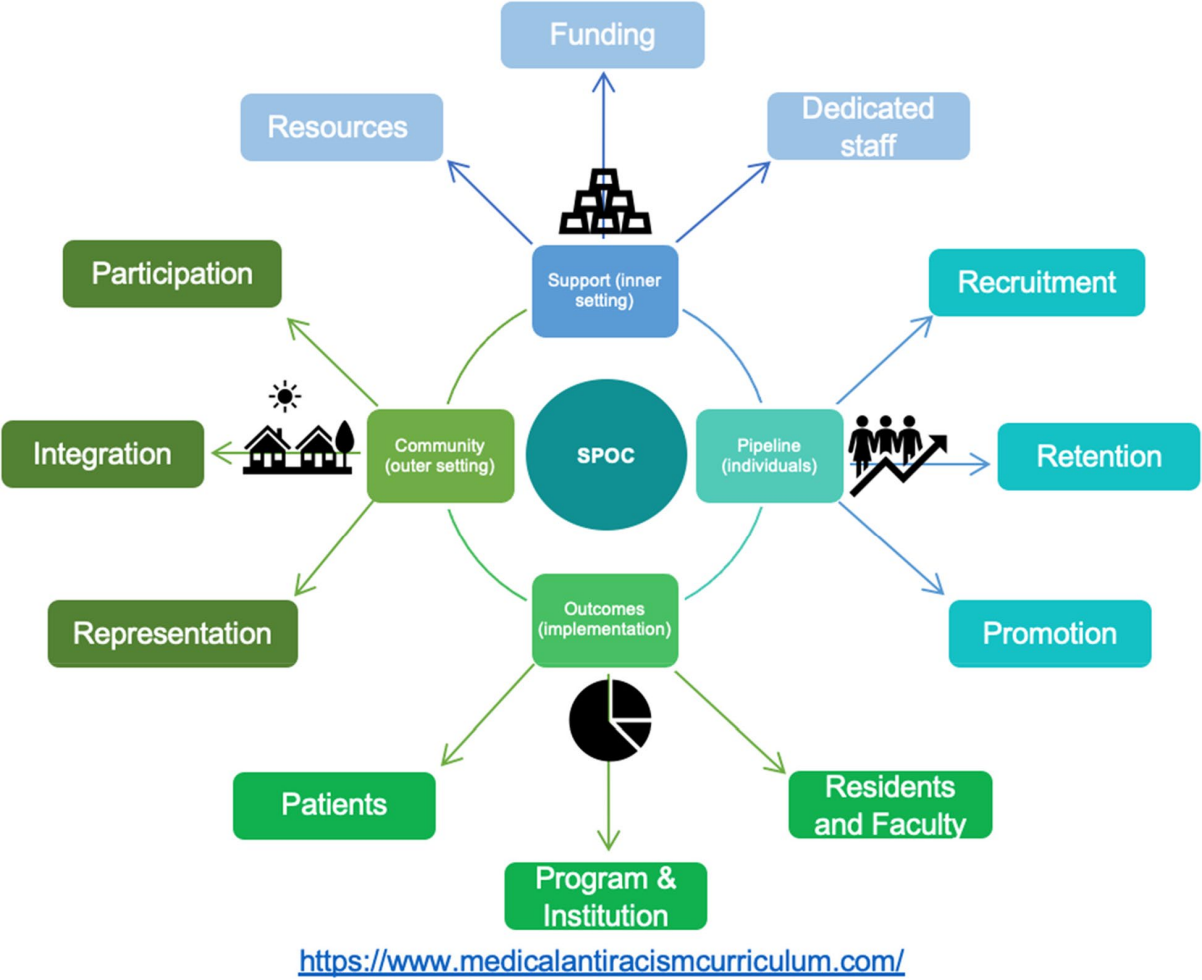
The SPOC (Support – Pipeline – Outcomes – Community) Model for creating sustainable anti-racism curricula for physicians in training

To develop a visual guide for programs designing sustainable anti-racism curricula in the future, the Consolidated Framework for Implementation Research (CFIR) [19–21] was used to organize themes and sub-themes derived from key informant interviews, and guide creation of the SPOC (Support – Pipeline – Outcomes – Community) Model (see Fig. 1) - pronounced “spoke”. Using the CFIR domains that would influence success of such curricula, the team contextualized themes and sub-themes derived from interviews, to design the model as an actionable guide. These CFIR domains considered included: the outer setting (e.g. society as a whole, policies and laws, and the hospital system), innovation (e.g. the curriculum structure, strategies, and resources), inner setting (e.g. university or college and program including resources and available funding), individuals (e.g. the teachers, learners, champions, and those the curriculum affects such as the patients), and the implementation process (e.g. what measured outcomes will strongly influence how successful the curricula are).

Integrating the reorganized themes and subthemes into the CFIR domains, we included as the four inner spokes of the model: (1) Support, (2) Pipeline, (3) Outcomes, and (4) Community. Within the first spoke of ‘Support’, programs must ensure that resources for the curriculum are secured, that funding is present for the activities that the curriculum will include, and finally that dedicated staff are identified, and importantly, compensated for their time spent building and maintaining the curriculum. As part of the second spoke, ‘Pipeline’, programs must ensure that recruitment, retention, and promotion of traditionally under-represented minorities in medicine occurs within their program. Within the third spoke of ‘Outcomes’, it is imperative that outcomes focused on residents, the program and institution itself, and last, but likely most importantly, on patients are agreed upon, tracked, and utilized to measure program success. If these metrics are not improving over time, the curriculum must be adjusted. Finally, the last spoke, ‘Community’, involves inclusion of the community the program serves in program planning and evaluation (e.g., community action boards), integration of the program into the community (e.g. community based rotations), and participation in community activities by residents and faculty (e.g., involvement in STEM programs at local schools).

## Discussion

### Summary of findings

This study employed in-depth interviews with key informants, formative research, and existing theoretical frameworks, to determine strategies for creating sustainable, effective, and continuously improving anti-racism curricula for medical residents. Findings highlight the need for focusing on ensuring the four key elements of (1) Support (2) Pipeline (3) Outcomes and (4) Community. The SPOC model can be used as a roadmap for future anti-racism curriculum design and implementation.

### Key messages

One of the key messages of this work is that 'what gets measured gets done', and can be used to hold people accountable, which is essential if anti-racism work is to be successful. Including outcome and accountability measures tied to credentialing and accreditation is necessary to ensure long-term goals are met and continuous quality improvement occurs. Recruitment and retention of underrepresented minorities into programs and leadership positions is also necessary and, along with resource allocation and availability, is essential to long-term sustainability of anti-racism curricula. Community involvement is also key.

### Limitations and strengths

Findings from this study are subject to serveral limitaitons. The formative and qualitative nature of the work potentially limits the generalizability of the findings. In addition, this work may be limited by regional or system restrictions (for example prohibition of the use of the term “diversity” in programs in Florida). Moreover, individual program sustainability will depend on individual funding and resource availability which may vary from program to program. Finally, only 15 of the 20 participants opted to answer the optional demographic survey, limiting our ability to analyze complete demographic data for our study sample.

The strength of this work is rooted in the breadth and depth of experience represented by the participants as well as the research team itself which consisted of a diverse group of clinical, psychology, and public health practitioners, faculty, and educators. Team members included those who identified as URM. Multiple members also have extensive experience in adult learning, direct patient care to populations which are vulnerable, and teaching and curriculum building for adult learners in the areas of equity, social determinants of health, gender studies, and trauma informed care. The research team also included members with expertise in use of theoretical frameworks.

## Conclusions

This work is one of the first to qualitatively examine the experiences and perspectives of key stakeholders involved or invested in creating and advancing anti-racism curricula. It highlights the importance of considering sustainability factors when creating, implementing, and evaluating anti-racism curricula for physicians in training.

### Next steps

Next steps for research in this area should focus on examining measurable and meaningful outcomes of anti-racism curricula to identify what strategies and approaches are most effective in bringing about the desired downstream effects for residents, programs, health care systems, communities, and the health care system as a whole.

Policy makers and academic medical residency leaders can access and use this work to drive recommendations for future policies aimed to support and advance anti-racism teaching in physician training programs specifically: (1) Implementation of credentialing designed to promote integration of anti-racism teaching and (2) Implementation of academic hospital system outcome metrics aimed at measuring equity that are tied to accreditation. Policy makers should also recognize that some regional policies might be impeding this work in specific parts of the country, and advocacy efforts to change such policies at the local and national level will be needed to ensure this work can be done effectively.

Community participation is a key to success. Programs undertaking this work should involve the communities they serve in each step of the process. Establishing a community action board may be an effective manner in which to accomplish community involvement, but other methods can also be used.

### Available tools for future work

Along with specific curriculum tools (https://www.medicalantiracismcurriculum.com/), the SPOC Model can assist teachers and learners who are interested in designing, implementing, and evaluating their own anti-racism curricula. This model may support future residency programs’ efforts to ensure sustainability and continuous quality improvement components are built into curricula, and resultant downstream improvements in health outcomes and community involvement occurs.

## Data Availability

The datasets generated and/or analyzed during the current study are not publicly available due concern for keeping identity of participants anonymous but may be provided in part from the corresponding author on reasonable request.
